# Clinical trial protocol for PanDox: a phase I study of targeted chemotherapy delivery to non-resectable primary pancreatic tumours using thermosensitive liposomal doxorubicin (ThermoDox®) and focused ultrasound

**DOI:** 10.1186/s12885-023-11228-z

**Published:** 2023-09-23

**Authors:** Laura Spiers, Michael Gray, Paul Lyon, Shivan Sivakumar, Noor Bekkali, Shaun Scott, Linda Collins, Robert Carlisle, Feng Wu, Mark Middleton, Constantin Coussios

**Affiliations:** 1 Department of Oncology, University of Oxford, Old Road Campus Research Building, Roosevelt Drive, Oxford, OX3 7DQ UK; 2 Oxford University Hospitals NHS Foundation Trust, Oxford, OX3 7LE UK; 3 NIHR Oxford Biomedical Research Centre, Oxford, UK; 4 Institute of Biomedical Engineering, University of Oxford, Marcella Wing, Botnar Research Centre, Old Rd, Headington, Oxford, OX3 7LD UK; 5 Nuffield Department of Anaesthetics, John Radcliffe Hospital, Oxford, OX3 7LE UK; 6 Department of Oncology, Oncology Clinical Trials Office (OCTO), University of Oxford, Oxford, UK; 7 Nuffield Department of Surgery, Churchill Hospital, Oxford, OX3 7LE UK

**Keywords:** Doxorubicin, Liposomes, Liposomal, FUS, Focused ultrasound, Hyperthermia, Pancreatic ductal adenocarcinoma, Targeted drug delivery, Therapeutic ultrasound, ThermoDox®

## Abstract

**Background:**

The dense stroma of pancreatic ductal adenocarcinomas is a major barrier to drug delivery. To increase the local drug diffusion gradient, high doses of chemotherapeutic agent doxorubicin can be released from thermally-sensitive liposomes (ThermoDox®) using ultrasound-mediated hyperthermia at the tumour target. PanDox is designed as a Phase 1 single centre study to investigate enhancing drug delivery to adult patients with non-operable pancreatic ductal adenocarcinomas. The study compares a single cycle of either conventional doxorubicin alone or ThermoDox® with focused ultrasound-induced hyperthermia for targeted drug release.

**Methods:**

Adults with non-resectable pancreatic ductal adenocarcinoma are allocated to receive a single cycle of either doxorubicin alone (Arm A) or ThermoDox® with focused ultrasound-induced hyperthermia (Arm B), based on patient- and tumour-specific safety conditions. Participants in Arm B will undergo a general anaesthetic and pre-heating of the tumour by extra-corporal focused ultrasound (FUS). Rather than employing invasive thermometry, ultrasound parameters are derived from a patient-specific treatment planning model to reach the 41 °C target temperature for drug release. ThermoDox® is then concurrently infused with further ultrasound exposure. Tumour biopsies at the targeted site from all patients are analysed post-treatment using high performance liquid chromatography to quantify doxorubicin delivered to the tumour. The primary endpoint is defined as a statistically significant enhancement in concentration of total intra-tumoural doxorubicin, comparing samples from patients receiving liposomal drug with FUS to free drug alone. Participants are followed for 21 days post-treatment to assess secondary endpoints, including radiological assessment to measure changes in tumour activity by Positron Emission Tomography Response Criteria in Solid Tumours** (**PERCIST) criteria, adverse events and patient-reported symptoms.

**Discussion:**

This early phase study builds on previous work targeting tumours in the liver to investigate whether enhancement of chemotherapy delivery using ultrasound-mediated hyperthermia can be translated to the stroma-dense environment of pancreatic ductal adenocarcinoma. If successful, it could herald a new approach towards managing these difficult-to-treat tumours.

**Trial registration:**

ClinicalTrials.gov Identifier: NCT04852367. Registered 21^st^ April 2022.

EudraCT number: 2019–003950-10 (Registered 2019)

Iras Project ID: 272253 (Registered 2019)

Ethics Number: 20/EE/0284.

**Supplementary Information:**

The online version contains supplementary material available at 10.1186/s12885-023-11228-z.

## Background

Cancer treatments continue to advance but the five year overall survival rate of patients with pancreatic ductal adenocarcinoma (PDAC) remains one of the lowest of all solid tumours, currently at 11% for all stages combined [1]. Nearly a third of patients die from local progression and improved control of the primary is correlated with survival and symptom control [2, 3]. New approaches to non-resectable pancreatic cancer treatment are important as, even with chemotherapy, only a fifth of patients are alive at 1-year post diagnosis [4]. One of the main reasons for this poor response is the tumour micro-environment. Dense stroma within PDACs acts as a physical barrier to drug diffusion, and high concentrations of hyaluronic acid raise interstitial pressure, reducing perfusion of the tumour by causing vascular collapse [5]. Addressing this by increasing the dose of systemic chemotherapy would exceed the maximum tolerated dose in other tissues, resulting in significant toxicity to patients. Alternative approaches have been explored, such as the role of enzymatic degradation of hyaluronic acid in the stroma in the phase III trial HALO-301. This failed to demonstrate improvement in Overall Survival (OS), Progression-Free Survival (PFS) or duration of response in patients with stage IV pancreatic cancer [6].

Despite its retroperitoneal position and proximity to major structures, the pancreas can be directly targeted with localised interventions such as ultrasound, and ablation of pancreatic tumours using High Intensity Focused Ultrasound (HIFU) has been performed safely [7]. This approach uses the thermal and cavitation effects of focused ultrasound (FUS) beams of typical intensity 1–20 kW/cm^2^ for direct tumour destruction by thermal ablation [8]. Lower spatial peak temporal average intensities (I_spta_) between 50-500W/cm^2^ may be used to induce smaller and more transient temperature elevations (~ 4 °C to 5 °C). Vasodilation of capillaries resulting from localised heating of the tumour by FUS facilitates drug delivery through steeper diffusion gradients, due to increased blood vessel permeability, enhanced diffusivity through tissue, and reduced interstitial pressure [9]. This approach maintains the non-invasiveness, precision, and real-time mapping advantages of FUS, whilst minimising excess heat effects on nearby healthy tissue, and is therefore suited to facilitate targeted intra-tumoral drug release [10, 11].

Several trials have combined FUS with systemically administered therapeutics. A phase I study in patients with unresectable pancreatic cancer combined gemcitabine (1000 mg/m^2^ as a 30-min intravenous infusion weekly for three weeks, followed by a one-week rest period) and FUS at sub-ablative thresholds (input power 2 kW/cm^2^), administered within 24 h of each gemcitabine dose. Three patients received the combination therapy, and this was well tolerated. The average time to progression was 11.6 months, vs 4.4 months in 9 patients receiving FUS only [12]. In a phase II trial, patients with unresectable pancreatic cancer received gemcitabine 1000 mg/m2 on day 1, 8 and 15 and FUS (input power 3 kW/cm^2^) on day 1, 3 and 5. The response rate was 43.6% and 2 of 37 cases had complete response. Overall survival (OS) at 12 months was 50.6% (95% CI, 36.7–64.5%). Treatment was well tolerated, with the most frequent toxicity being myelosuppression [8].

The drug itself can be optimised for use with FUS. ThermoDox® (Celsion Corporation, USA) is approved for investigational use and consists of the anthracycline chemotherapeutic, doxorubicin, encased in a thermosensitive lyso-liposome with PEGylation to increase its half-life. Mild hyperthermia (39.5—42 °C) causes instantaneous chemotherapy release from the long-circulating Lyso-Thermosensitive Liposomal Doxorubicin (LTSD) [13]. This gives spatial and temporal control of drug release by targeted tumour heating. The DNA intercalation properties of doxorubicin means that a proportion of drug remains in tissue rather than being reabsorbed back in to the microcirculation [14]. Additionally, it is easily detectable by fluorescent imaging [15]. These properties make ThermoDox® a good candidate for assessing drug delivery by FUS-induced hyperthermia. In murine models of PDAC, ThermoDox® was delivered using HIFU with magnetic resonance (MR-HIFU) for thermal monitoring of treatment. The tumour drug concentration administered with MR-HIFU was 23 times greater than LTSD delivered alone [16]. In the clinical setting, a first-in-human study (TARDOX, NCT02181075) used extracorporeal FUS coupled to a B-mode US system. The aim of the TARDOX trial was to assess safety and feasibility of drug release ThermoDox® to tumours in the liver. It demonstrated that the combination of FUS and ThermoDox® is safe, feasible and resulted in a 3.7-fold average increase in intra-tumoural drug concentration, for the same overall systemic dose of the drug. Localised radiological tumour responses confined to the FUS-exposed region alone were demonstrated after a single treatment cycle [10].

PanDox (NCT04852367) is a phase I study to investigate the safety, applicability and usefulness of this approach to pancreatic tumours. In the absence of any invasive or non-invasive thermometry, the ultrasound treatment is delivered based on a personalised therapeutic ultrasound treatment plan. This builds on the validated TARDOX treatment planning model, constructed using inputs of anatomical data from computerised tomography (CT) and magnetic resonance imaging (MRI), along with acoustic and thermal properties for the constituent tissues. The model can then produce acoustic pressure and temperature maps of the target area, and generate personalised ultrasound treatment plans (power, duty cycle, and therapeutic treatment volume) [17]. An important outcome from the TARDOX trial was that patients treated using a modelling-only approach experienced comparable enhancements in drug delivery to those treated with invasive thermometry. This gives confidence to using the non-invasive approach in PanDox, but some modifications to the model are required to compensate for the anatomical differences in treating pancreas rather than liver. This has required the full characterization under a separate ethics application through the Oxford Radcliffe Biobank research tissue bank (reference 9/SC/0173) of the until this point unknown acoustic properties of human pancreatic tumours, spleen and duodenum, and are described elsewhere [18].

## Methods/design

### Aim

The primary aim is to quantify the enhancement of doxorubicin concentration delivered to pancreatic tumours, for a given systemic dose. It compares delivery of intravenous free doxorubicin alone to targeted drug release of liposomal doxorubicin (ThermoDox®) by localized hyperthermia induced by an extracorporeal ultrasound-guided FUS device.

### Setting

PanDox is a phase I prospective non-randomised safety cohort study with all patients recruited from a single UK site. Participants must have confirmed non-resectable (locally advanced or metastatic) pancreatic ductal adenocarcinoma and may have received previous chemotherapy.

### Design

The study has an open label design with all participants receiving a single dose of either standard doxorubicin or systemic ThermoDox® with ultrasound-guided FUS targeted at a single pancreas tumour using the Model JC-200 Focused Ultrasound Tumour Therapeutic System (Haifu Technology Company, Chongqing, China), which is clinically approved (CE-marked) for tumour therapy in Europe and China. The single cycle design is for minimal impact on participant’s care pathway, with treatment delivery planned during the window of opportunity between consenting to and commencing standard chemotherapy.

**Fig. 1:**
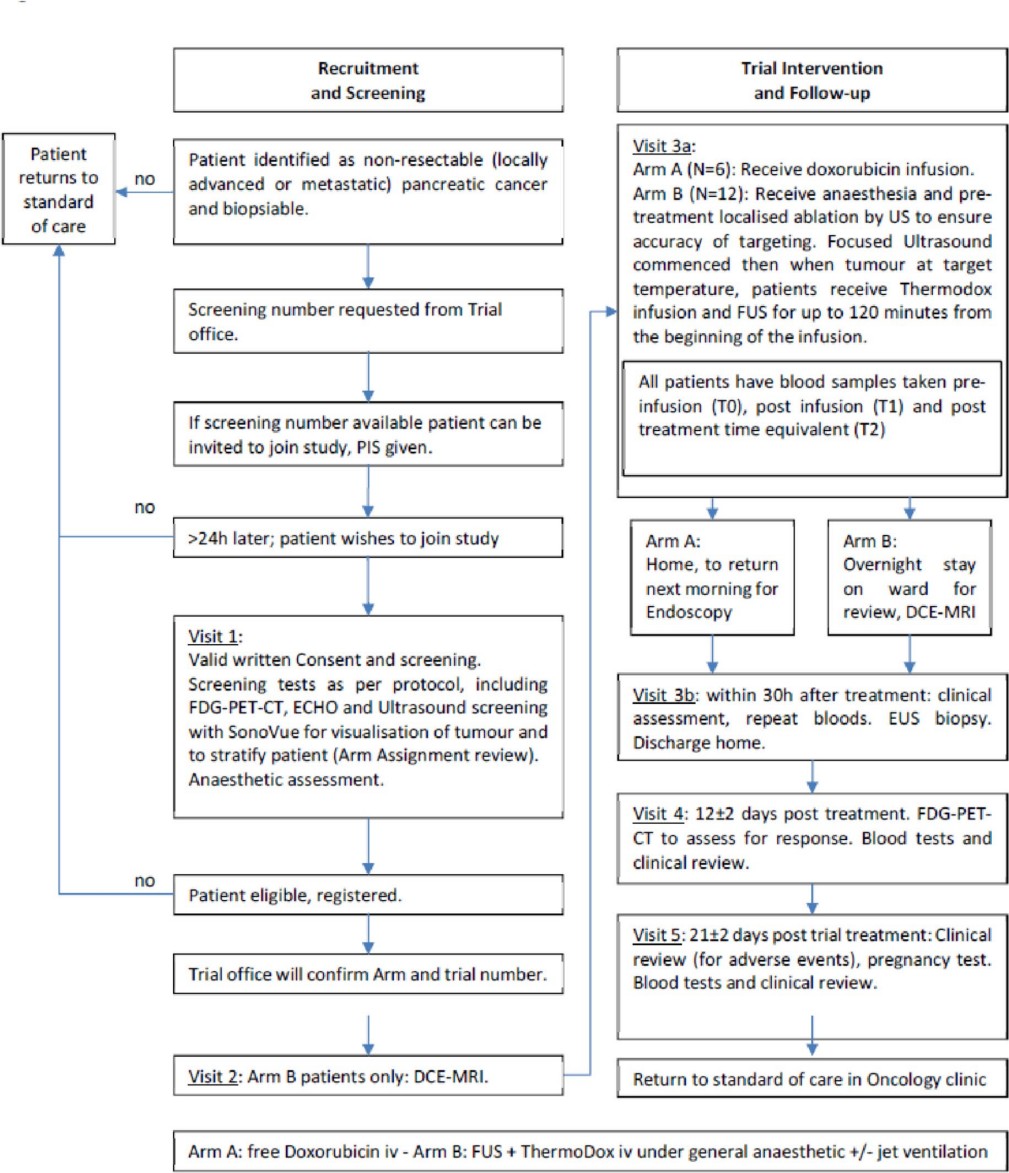
PanDox trial design overview

The trial pathway for patients is summarised in Fig. 1.

### Screening

Patients for consideration must have cross-sectional imaging review for tumour targeting suitability, and MDT (Multi-Disciplinary Team) agreement before being approached.

Screening comprises clinical history and examination, routine blood tests, cardiac assessment by ECG (Electrocardiogram) and ECHO (Echocardiogram), and a pre-operative assessment to ensure suitability for general anaesthetic. A full checklist is in supplementary material 1. After initial selection using cross-sectional imaging, with patient consent, the suitability of potential target lesions is assessed based on ultrasound abdominal examination. Further radiological assessment by fluorodeoxyglucose positron emission tomography (18F-FDG-PET), CT and MRI allows for baseline comparison as well as providing inputs for treatment planning as described above. (Table 1)

**Table 1:** Clinically acceptable laboratory results during screening window

**Lab Test**	**Value required**
Haemoglobin (Hb) (transfusion to achieve this allowed)	≥ 9 g/dL
Neutrophils	≥ 1.5 10^9^/L
Platelet count	≥ 100 10^9^/L
ALT	≤ 2.5 × ULN
Alkaline phosphatase	≤ 5 × ULN
Serum Bilirubin (stenting to achieve this allowed)	≤ 1.5 × ULN
Creatinine Clearance (Calculated by Cockcroft-Gault criteria)	≥ 50 ml/min
International Normalised Ratio (INR)	< 1.5 unless taking oral anticoagulant (this to be stopped at least 1 week prior to biopsy, at which point this INR limit will then apply)

### Characteristics of participants

#### Inclusion criteria

The following eligibility criteria apply:

1. Able to give informed consent prior to any screening procedures being performed and is able and willing to comply with the protocol and its requirements.
2. Male or Female, aged 18 years or above.
3. Prior histological confirmation of pancreatic adenocarcinoma
  Non-resectable or metastatic (stage IV)
  The primary pancreatic lesion measuring at least 1.5 cm minimum diameter and amenable to EUS (endoscopic ultrasound) biopsy sampling
4. Eastern Co-operative Oncology Group (ECOG) performance status 0–1
5. Left ventricular ejection fraction (LVEF) ≥ 50% as determined by echocardiogram
6. Willing to allow his or her General Practitioner and Consultant, if appropriate, to be notified of participation in the trial.
7. Life expectancy of at least 3 months
8. Female participants of childbearing potential and male participants whose partner is of childbearing potential must be willing to ensure that they or their partner use highly effective contraception during the trial and for 6 months thereafter.
9. Participant has clinically acceptable laboratory results during screening window:

#### Exclusion criteria

A patient is ineligible for inclusion in this study if any of the following criteria apply.

1. Significant renal or hepatic impairment.
2. Unstable ischemic heart disease, cardiac dysrhythmias, coronary/peripheral artery bypass graft or cerebrovascular accident within 6 months prior to starting treatment
3. Uncontrolled arterial hypertension despite medical treatment.
4. Ongoing congestive heart failure or cardiac dysrhythmias of National Cancer Institute Common Terminology Criteria for Adverse. Events (NCI CTCAE) Grade ≥ 2 or uncontrolled atrial fibrillation.
5. Previous myocardial infarction or acute inflammatory heart disease
6. On-going significant infection (chest, urine, blood, intra-abdominal).
7. Uncontrolled diabetes.
8. Scheduled elective surgery or other procedures requiring general anaesthesia during the trial.
9. Patients who have undergone major surgery ≤ 4 weeks prior to starting study drug or who have not recovered from side effects of such procedure
10. Previous targeted therapies to the pancreatic adenocarcinoma (including radiofrequency ablation or radiotherapy)
11. History of other malignancy less than 3 years before the diagnosis of current cancer, EXCLUDING the following: non-melanoma skin cancer, in situ carcinoma of the cervix treated surgically with curative intent, other malignant tumours that have been treated curatively and patient is deemed disease-free
12. Endocrine therapy – patients with prostate cancer may continue to receive endocrine therapy to maintain castrate levels of androgens
13. Known allergic reactions to any of the drugs or liposomal components or intravenous imaging agents used in this study
14. Resting electrocardiogram (ECG) with QTc > 480ms at 2 or more time points within a 24h period (using Fredericia correction).
15. Other severe acute or chronic medical or psychiatric conditions or laboratory abnormalities that the investigator considers would make the patient a poor trial candidate, would impart excess risk associated with study participation or drug administration or could interfere with protocol compliance or the interpretation of trial results.
16. Female participant who is pregnant, lactating or planning pregnancy during the trial. However, those female patients who have a negative serum pregnancy test before enrolment and agree to use one highly effective form of contraception in addition to condom plus spermicide, for four weeks before entering the trial, during the trial and for six months afterwards are considered eligible.
17. Male patients with partners of child-bearing potential unless they agree to take measures not to father children by using one form of highly effective contraception during the trial and for six months afterwards. Men with pregnant or lactating partners should be advised to use barrier method contraception during the trial and for six months afterwards to prevent exposure to the foetus or neonate.
18. Participants who have participated in another research trial involving an investigational product in the past 12 weeks.
19. Severe immunologic defect or compromised bone marrow function.
20. Patients who are serologically positive for Hepatitis B, Hepatitis C or Human Immunodeficiency Virus (HIV).
21. Previous doxorubicin and epirubicin must not have exceeded 450 mg/m^2^ and 900 mg/m^2^, respectively.
22. Patients who have a contraindication to MRI scans, for example patients who have a cardiac pacemaker, will be excluded from Arm B as per Arm Assignment criteria, (Supplementary material 2).

### Allocation

Participants who successfully complete screening are allocated to either Arm A (doxorubicin) or Arm B (ThermoDox® with FUS). Given the paucity of patients at the appropriate stage of disease to meet inclusion criteria without impacting their own care pathway, patients are not randomized but assigned to either arm, based on a pre-determined checklist (supplementary material 2). This is to ensure feasibility and safety of patients to undergo FUS and considers tumour factors such as tumour location, proximity to vessels, overlying gastro-intestinal tract and patient factors or contra-indications to MRI or general anaesthetic. Inclusion of patients for whom it is felt FUS therapy is not suitable enables these participants who have successfully completed screening to still take part in a clinical trial (with the benefits this may bring) and strengthens the trial design by having a group of patients who have been exposed to the drug but not FUS.

Participants in Arm B undergo Dynamic Contrast-Enhanced Magnetic Resonance Imaging (DCE-MRI) as part of a FUS safety assessment. Comparison with repeat imaging post-treatment will demonstrate any off-target ablation.

### Treatment

Patients in Arm A receive pre-medications as per local practice, followed by a single intravenous dose 50 mg/m^2^ Doxorubicin in 250 mL normal saline over a 30 min intravenous infusion.

**Fig. 2:**
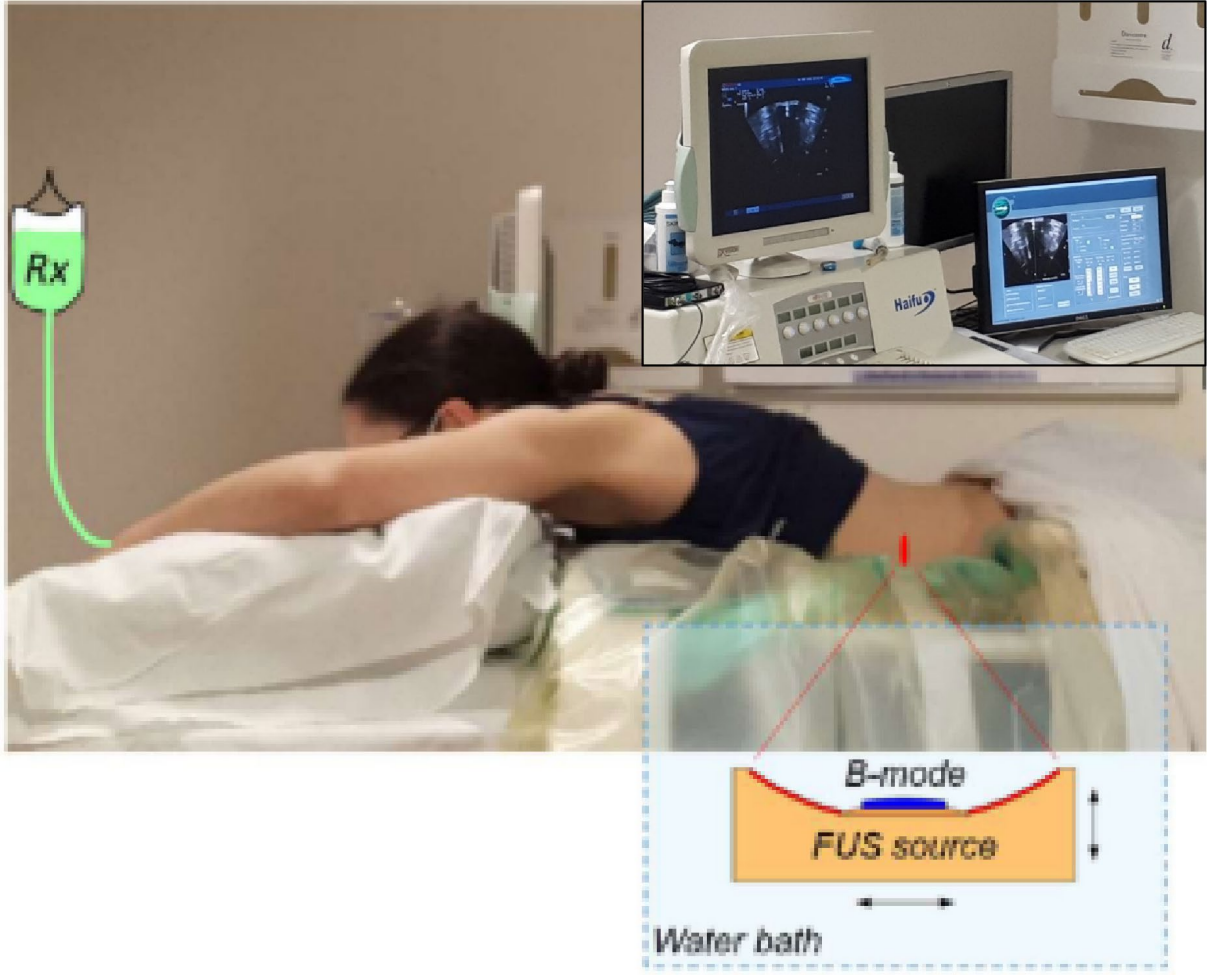
The PanDox FUS treatment concept. The prone patient lies over the JC200 water bath containing a FUS source fitted with a coaxial B-mode probe. For induction of mild hyperthermia, the FUS source is continuously scanned under B-mode guidance (inset) to cover the prescribed treatment volume

Patients in Arm B are positioned as per their planning session. This is most likely lying prone on the bed of the JC-200 ultrasound-guided system, with the patient’s abdomen in contact with a degassed and temperature-controlled water bath underneath, to couple the ultrasound field to the patient (Fig. 2).

Within the waterbath sits a fixed focus single element high power source (“FUS source”, 200-mm diameter, 0.96 MHz, generating a focus with a transverse 3-dB beam-width of 1.2 mm and an axial 3-dB length of 9.5 mm) with a coaxially configured curvilinear imaging transducer (“B-mode”) for simultaneous guidance and treatment (JC200 Catalogue, 2023). A degassed water balloon within the bath may be deployed to optimise the beam path between transducers and patient. The tumour is identified using B-mode imaging with the patient awake. Urinary catheterisation and placement of a de-gassed naso-gastric tube is followed by supine induction of general anaesthesia. General anaesthesia with high-frequency jet ventilation (HFJV) facilitates FUS imaging and treatment by minimising respiratory-induced motion of the target tumour. The patient is returned to the prone position over the waterbath for final mapping of the treatment volume, again under B-mode guidance. Application of HIFU for tiny foci of tumour ablation helps confirm location targeting accuracy, visualised by B-mode grayscale intensity increase within the target and may give a treatment reference point for Endoscopic Ultrasound (EUS) biopsy. No more than two ablation spots are applied, to minimize the risk of any observed effects being due to ablation rather than drug delivery. After confirming the target, lower-intensity FUS is moved through the tumour volume to raise the bulk tumour temperature above the thermal release threshold. No invasive thermometry will be used. Instead, an individualised plan of FUS parameters (power, duty cycle, scanning speed, unit spacing), is dependent on patient and tumour anatomy and devised from patient imaging and computer modelling [17, 18]. Upon reaching the optimal tumour temperature, a single cycle of 50 mg/m^2^ ThermoDox® in 250 mL 0.9% saline is infused intravenously over 30 min. Concurrently, the focus of the FUS device continues to be moved across the target tumour to maintain a volume of up to 125cm^3^ in the range 39.5 ^o^C to 43 ^o^C to mediate drug release. FUS continues following infusion, for up to 120 min from the start of infusion when peak circulating drug levels are highest, as based on pharmacokinetic data from the manufacturer [13].

### Post-treatment

Patients are reviewed for adverse events related to drug and ultrasound (where appropriate). Arm A patients may go home after treatment and return for clinical review and blood tests the morning after treatment, before EUS biopsy.

Arm B patients may be admitted to the Oncology ward for overnight observation following review. They will undergo DCE-MRI within 30 h post-treatment.

Within 30 h after therapy, all patients will undergo EUS biopsy for pancreatic tumour sampling (up to 5 core biopsies). Three samples will be analysed for primary endpoint (intra-tumoural doxorubicin) and where remaining samples allow for exploratory endpoint analysis of doxorubicin intercalation.

After 14 days post-treatment, patients undergo clinical review for adverse events and ^18^F-FDG-PET-CT to assess tumour size and activity. Impact on patient-reported symptoms is assessed by questionnaire.

Patients are reviewed at day 21 to again assess for adverse events. Care then transfers back to local Oncology teams, with follow-up of any adverse events as needed.

### Sample size

The PanDox study is intended to recruit 18 evaluable participants. This sample size reflects that this is a Phase I study and is in line with the available funding resources and likely recruitment rates. Using estimates of mean tissue doxorubicin and standard deviation from the TARDOX trial [10], the participant number is predicted to be large enough to demonstrate statistically meaningful enhanced drug delivery using an unpaired t-test. Additionally, the sample size is small enough to ensure that participants are not unnecessarily recruited and exposed to potential side effects.

### Analysis plan

The primary outcome measure is the concentration of intra-tumoural doxorubicin at the targeted tumour site, comparing the average in biopsy samples from Arm A patients (drug only) to Arm B (drug and FUS). All participants who received an intervention will be included in the primary endpoint analysis, thus evaluated on an intention to treat basis. To be included in this analysis, intra-tumoural drug concentrations from biopsy samples must have been successfully analysed using a Good Laboratory Practice-validated Liquid Chromatography with tandem mass spectrometry (LC/MS/MS) assay, based on previously published methods [19]. We would hope to have up to three samples per patient, to help mitigate tumour heterogeneity. To satisfy the primary endpoint, demonstration of a statistically significant enhancement in concentration of total intra-tumoural doxorubicin from tumour biopsies at the targeted tumour site is required, comparing samples from patients receiving drug with FUS compared to drug alone. The significance levels used will be 0.05, and 95% confidence intervals will be reported.

Secondary endpoints relate to adverse event monitoring, performed for 21 days post-intervention and consist of clinical, haematological and biochemical review. Adverse events are assessed for expectedness and causality to the drug and to FUS, and classified according to the NCI CTCAE, version 5.0. Expected effects of doxorubicin include bone marrow suppression (in particular neutropenia and thrombocytopenia), and changes to liver enzymes due to hepatic elimination. Symptoms will be monitored by a pancreatic cancer-specific patient questionnaire (EORTC QLQ—PAN26) throughout each patient’s participation. Radiological responses of tumour volume and activity (as measured by maximum standardized uptake value, SUV_max_) will be evaluated by comparison of ^18^F-FDG-PET-CT images at baseline and 21 days using PERCIST criteria in the target tumour alone.

Tertiary (exploratory) end points relate to doxorubicin effects on the tumour biopsy samples (quantification of doxorubicin metabolites, and visualisation of doxorubicin intercalation), and whether an effect on tumour markers is seen. Doxorubicinol, Doxorubicinone, Doxorubicinolone and 7-Deoxy Doxorubicin Aglycone are measured by Liquid Chromatography with tandem mass spectrometry (LC/MS/MS) to Good Laboratory Practice standards, and are included to capture doxorubicin metabolism from hyperthermia or due to time lapse between treatment and sampling [19].

Microscopy for doxorubicin visualisation is performed where tissue allows, after prioritising tumour sampling for the primary endpoint. Tissue is embedded in optimum cutting temperature medium, cut into 5 µm thick sections every 100 µm and stained with DAPI (for nucleus visualisation) and immunohistochemistry for CD31 (endothelium) performed. Confocal fluorescence microscopy is used to visualise doxorubicin co-localisation with DAPI and vasculature, based on previous protocols [20, 21].

Plasma samples are collected immediately before the start of ThermoDox® infusion, immediately after completion of ThermoDox® infusion, and immediately after completion of FUS exposure to evaluate doxorubicin pharmacokinetics.

Tumour marker CA19-9 is used in the clinic to monitor treatment response and would be checked at commencement of chemotherapy (whether first or subsequent lines) as standard practice.

## Discussion

This study design allows the “drug and device” approach to enhancing delivery to be trialled with minimum impact upon the treatment pathway for patients with non-resectable pancreatic cancer. The single-cycle treatment will be delivered either within the window of initiating next line chemotherapy or for patients who have exhausted standard therapy options. Arm A facilitates inclusion of patients for whom it is felt FUS therapy is not suitable enabling these participants to still take part in a clinical trial (with the benefits this may bring) and strengthens the trial design by having a group of patients who have been exposed to the drug but not FUS. ThermoDox® alone is not used as the comparator based on studies of animals models of pancreatic cancer that demonstrated limited drug delivery compared to conventional doxorubicin [16].

A close collaborative approach is required to co-ordinate Oncology, Radiology, Anaesthetic and Endoscopy departments to deliver the trial meeting the specific timelines. The protocol is flexible to allow tumour biopsy by radiological means if an EUS approach is not available, appropriate or initially successful. Initiating treatment quickly is particularly important for this patient group, as PDACs are aggressive and patients can decondition within a short number of weeks.

If this early phase study can demonstrate that ultrasound-mediated hyperthermia can safely and effectively enhance the delivery of doxorubicin in this difficult-to-treat tumour type, it could pave the way for utilising a range of anti-cancer therapies in combination with FUS-induced hyperthermia for patients with pancreatic cancer. Moreover, the ability to provide this therapy without the need for invasive or MR-based thermometry holds the potential to greatly expand accessibility and throughput.

### Supplementary Information

**Additional file 1: Supplementary material 1.** schedule of events

**Additional file 2: Supplementary material 2.** Arm allocation checklist

**Additional file 3: Supplementary material 3.** CONSORT flow diagram

## Data Availability

No applicable data. A description of the trial and its design are available at clinicaltrials.gov.

## Abbreviations

^18^F-FDG- PET: Fluorodeoxyglucose positron emission tomography
ALT: Alkaline Transaminase
CT: Computerised tomography
DCE-MRI: Dynamic Contrast-Enhanced Magnetic Resonance Imaging
ECG: Electrocardiogram
ECHO: Echocardiogram
ECOG: Eastern Co-operative Oncology Group
EUS: Endoscopic Ultrasound
FUS: Focused ultrasound
GLP: Good Laboratory Practice
Hb: Haemoglobin
HFJV: High Frequency Jet Ventilation
HIFU: High Intensity Focused Ultrasound
HIV: Human Immunodeficiency Virus
HPLC: High performance liquid chromatography
INR: International normalised ratio
I_spta_: Spatial peak temporal average intensities
LVEF: Left ventricular ejection fraction
LTSD: Lyso-Thermosensitive Liposomal Doxorubicin
MDT: Multi-Disciplinary Team
MR-HIFU: Magnetic resonance with HIFU
MRI: Magnetic Resonance Imaging
NCI CTCAE: National Cancer Institute Common Terminology Criteria for Adverse. Events
OS: Overall Survival
PDAC: Pancreatic Ductal Adenocarcinoma
PERCIST: Positron Emission Tomography Response Criteria in Solid Tumours
PFS: Progression-Free Survival
SUV_max_: Maximum standardized uptake value
