# Supplementary material for: Clinical trial protocol for PanDox: a phase I study of targeted chemotherapy delivery to non-resectable primary pancreatic tumours using thermosensitive liposomal doxorubicin (ThermoDox®) and focused ultrasound
Source: BMC Cancer. 2023 Sep 23;23:896. doi: 10.1186/s12885-023-11228-z (PMC10517508; doi:10.1186/s12885-023-11228-z)
Supplement: Supplementary file 1 — Additional file 1: Supplementary material 1. schedule of events [file 12885_2023_11228_MOESM1_ESM.pdf]

Supplementary material 1: schedule of events

Visit Description	Screening	Baseline DCE-MRI	Treatment	Post Treatment	Follow Up Visit	End of study
Visit number	1	2 **(Arm B only)**	3a	3b	4	5
Assessment window	-14 days	Post-registration but prior to treatment	Day 1	Day 2	Day 12 (+/- 2)	Day 21 (+/-2)
Informed Consent	x					
Inclusion/Exclusion criteria	x					
Demographics	x					
Medical History	x					
Concomitant medication	x				x	x
ECOG Performance Status	x				x	x
Physical examination	x			x	x	x
Anaesthetic assessment	x					
FUS Feasibility and Planning with Ultrasound	x					
Vital signs	x		x	x		
Height	x					
Weight	x					
Pregnancy Test	x					x
12 lead ECG	x					
ECHO	x					
Blood tests – Group and Save	x					
Haematology	x		x	x	x	x
Biochemistry	x		x	x	x	x
Coagulation studies	x					
Serology Assessments	x					
Tumour markers	x		x		x	x
QoL questionnaires – PAN26	x		x	x	x	x
Arm Assignment Review	x					
Radiological assessment - CT Chest/Abdo/Pelvis with contrast	x					
Radiological assessment - FDG-PET	x				x	
Trial Registration	x					
Radiological assessment of tumour(s) by DCE-MRI - **Arm B only**		x		x		
Pre-medication (20mg oral dexamethasone) **– Arm B only**		x (24h prior to FUS)				
Pre-medication (including for anaesthetic, anti-hypersensitivity and anti-emetics)			x			
Plasma samples – (T0)			x			
Plasma samples (T1)			x			
Plasma samples (T2)			x			
Dosing with Doxorubicin/ThermoDox + FUS			x			
Overnight observation - **Arm B only**			x			
Biopsy for tumour samples				x		
Adverse event recording			x	x	x	x
Serious Adverse Event Recording	x	x	x	x	x	x
