# Supplementary material for: Clinical trial protocol for PanDox: a phase I study of targeted chemotherapy delivery to non-resectable primary pancreatic tumours using thermosensitive liposomal doxorubicin (ThermoDox®) and focused ultrasound
Source: BMC Cancer. 2023 Sep 23;23:896. doi: 10.1186/s12885-023-11228-z (PMC10517508; doi:10.1186/s12885-023-11228-z)
Supplement: Supplementary file 2 — Additional file 2: Supplementary material 2. Arm allocation checklist [file 12885_2023_11228_MOESM2_ESM.pdf]

**Supplementary material 2: Arm Allocation checklist**

During screening, all patients are reviewed using the criteria below to assign to treatment group.

If any answer is "No" (shaded box) then the participant will be allocated to Arm A (standard doxorubicin only), otherwise to Arm B (ThermoDox® with FUS).

| **Characteristic** | **Yes** | **No** |
|---|---|---|
| **Patient** | | |
| BMI below 35 | | |
| Suitable for GA +/- jet ventilation | | |
| | | |
| **Tumour** | | |
| Tumour visible on US with patient in prone position | | |
| Distance from tumour to the stomach , duodenum, colon >1 cm | | |
| | | |
| **Clinical Reason** | | |
| Please confirm that no clinical reasons have been identified that would prevent the patient being assigned Arm B treatment. | | |
