# Supplementary figures and images for: Clinical trial protocol for PanDox: a phase I study of targeted chemotherapy delivery to non-resectable primary pancreatic tumours using thermosensitive liposomal doxorubicin (ThermoDox®) and focused ultrasound
Source: BMC Cancer. 2023 Sep 23;23:896. doi: 10.1186/s12885-023-11228-z (PMC10517508; doi:10.1186/s12885-023-11228-z)

Supplementary material 3: CONSORT flow diagram

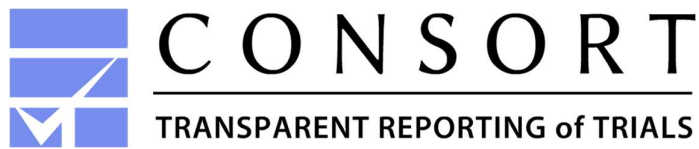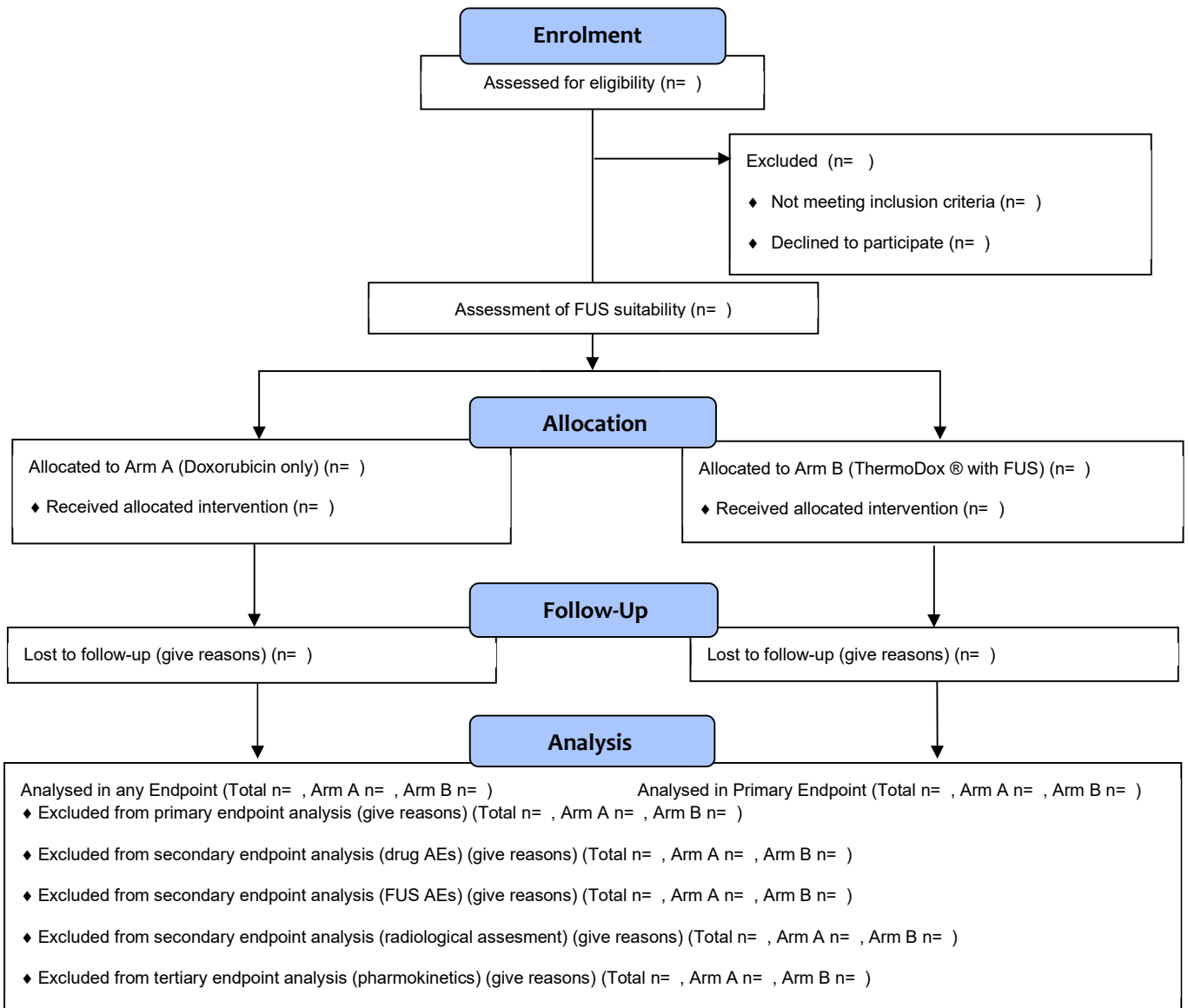

Supplement: Supplementary file 3 — Additional file 3: Supplementary material 3. CONSORT flow diagram [file 12885_2023_11228_MOESM3_ESM.pdf]
